# Oleanolic acid improves the *in vitro* developmental competence of early porcine embryos by reducing oxidative stress and ameliorating mitochondrial function

**DOI:** 10.5713/ab.24.0307

**Published:** 2024-08-26

**Authors:** Yan-Wei Dong, He-Xuan Qu, Yan-Qiu Wang, Jia-Jia Qi, Hua-Kai Wei, Bo-Xing Sun, Hao Sun, Shuang Liang

**Affiliations:** 1 Department of Animals Sciences, College of Animal Sciences, Jilin University, Changchun130062, China

**Keywords:** Mitochondrial Function, Oleanolic Acid, Oxidative Stress, Porcine Early Embryos

## Abstract

**Objective:**

Oleanolic acid (OA) is a pentacyclic triterpenoid with antioxidant activity that can be an effective scavenger of free radicals in cells. This study was designed to investigate the effects of OA on porcine early embryo developmental competence *in vitro* and its possible mechanisms of action.

**Methods:**

In the present study, parthenogenetically activated porcine embryos were used as models to assess the effects of OA on the *in vitro* developmental capacity of early porcine embryos *in vitro*. Zygotic genome activation, mitochondrial function, oxidative stress, cell proliferation and apoptosis in early porcine embryos were examined after supplementing the culture medium with 5 μM OA.

**Results:**

The results showed that 5 μM OA supplementation not only significantly increased the blastocyst diameter in early embryos on day 6 but also increased the total cell number of blastocysts. Furthermore, OA supplementation increased the blastocyst proliferation rate and decreased blastocyst apoptosis. Moreover, OA supplementation significantly increased the proportion of embryos that developed to the 4-cell stage after 48 h of *in vitro* culture and upregulated the expression of genes associated with zygotic genome activation (*DPPA2* and *ZSCAN4*). Notably, OA alleviated oxidative stress by reducing the intracellular levels of reactive oxygen species and increasing the intracellular levels of reduced glutathione at the 4-cell stage and increased the activities of superoxide dismutase and catalase. Concurrently, OA significantly increased the mitochondrial membrane potential and intracellular adenosine 5’-triphosphate content.

**Conclusion:**

These results suggest that OA promotes the *in vitro* developmental competence of parthenogenetically activated porcine embryos by reducing oxidative stress and improving mitochondrial function during *in vitro* culture and that OA may contribute to the efficiency of *in vitro* embryo production.

## INTRODUCTION

*In vitro* embryo culture is fundamental in animal embryo engineering and an important prerequisite for gene editing technology [1]. Therefore, it is crucial that high-quality *in vitro* embryos are available for the development of technologies such as embryo transfer [2], animal cloning [3], and transgenic animal development [4].

Under normal physiological conditions, the reactive oxygen species (ROS) generated via metabolism during embryonic development can be scavenged by the body’s antioxidant defense mechanisms, thus maintaining a dynamic balance of intracellular ROS [5]. However, during *in vitro* culture, early embryos are susceptible to external environmental factors that disrupt intracellular redox homeostasis, leading to ROS accumulation and oxidative stress. ROS can cause embryonic arrest by inducing DNA damage [6], lipid peroxidation [7], and mitochondrial damage [8]. Previous studies have shown that supplementing the culture media with exogenous natural antioxidants can effectively increase the antioxidant capacity of embryos and improve embryonic development competence [9–11]. Laminarin and asiatic acid were shown to attenuate H_2_O_2_-induced ROS production in parthenogenetically activated porcine embryos, whereas lycopene was found to significantly diminish intracellular ROS levels and reduce apoptosis in fertilized porcine embryos *in vitro* [9–11]. Furthermore, asiatic acid promotes early embryo development after both parthenogenetic activation (PA) and *in vitro* fertilization (IVF) and elevates the expression of antioxidant genes after somatic cell nuclear transfer (SCNT) embryos.

Oleanolic acid (OA) is a natural antioxidant found in many plants that has antioxidant, anticancer and anti-inflammatory properties [12]. Previous research has shown that OA can effectively alleviate oxidative stress by increasing the expression of antioxidant enzymes such as glutathione peroxidase and superoxide dismutase (SOD) by regulating Nrf2 in rat liver microsomes [13]. In addition, by activating the Nrf2 signaling pathway, OA regulates cell proliferation and apoptosis and reduces oxidative stress-mediated toxicity in the liver [14]. Another study showed that OA exerts antioxidant effects in PC12 neuronal cells, as evidenced by significant increases in glutathione (GSH) levels and catalase (CAT) and SOD activities [15]. Gutierrez et al. reported that OA prevents intestinal lipid peroxidation and superoxide anion accumulation and induces the expression of the ROS scavenger sestrin-3 [16].

Polyspermy poses a significant challenge in the creation of *in vitro* models of early embryos. Under normal *in vivo* conditions, the probability of polyspermy is less than 5%; however, this risk increases during IVF processes [17]. Several factors contribute to this increase, including the quality of the oocyte, the viability and morphology of the sperm, and the composition of the fertilization medium, which can cause considerable complications in experimental research [18]. In light of these limitations, parthenogenetic embryos have emerged as promising alternative embryological models. They have been observed to develop in a manner closely resembling the development of the normal embryos of various animals [19], offering a valuable tool for research.

Therefore, we hypothesized that supplementing the culture media with OA could alleviate the embryonic damage induced by oxidative stress during *in vitro* embryonic development. In this study, we used parthenogenetically activated porcine embryos as models to analyze the effects of OA on the development of early porcine embryos in terms of zygotic genome activation, antioxidant capacity, mitochondrial function, and embryonic cell proliferation and apoptosis. This provides a theoretical basis by which high-quality early embryos can be obtained and the embryo *in vitro* culture system can be further optimized.

## MATERIALS AND METHODS

### Reagents

All reagents and chemicals were supplied by Sigma-Aldrich (St. Louis, MO, USA) unless otherwise noted.

### Porcine oocyte collection and *in vitro* maturation

The porcine ovaries used in the experiment were obtained from local abattoirs, placed in saline at 35°C to 37°C and transported to the laboratory within 2 h. Cumulus-oocyte complexes (COCs) were extracted from 3 to 6 mm long follicles using a disposable 10 mL syringe, after which they were washed three times with 4-(2-Hydroxyethyl)piperazine-1-ethanesulfonic acid buffer. COCs containing three or more layers surrounded by oocytes with a homogeneous cytoplasm were selected under a stereomicroscope (S22-LGB; Nikon, Shanghai, China). Then, the chosen COCs were transferred to TCM-199 medium (supplemented with 10 IU/mL luteinizing hormone, 10 IU/mL follicle stimulating hormone, 0.91 mM sodium pyruvate, 10 ng/mL epidermal growth factor, 0.6 mM L-cysteine, 75 μg/mL kanamycin and 10% porcine follicular fluid) and covered with mineral oil. There were 50 to 60 COCs in 500 μL of maturation medium per well. The COCs were then incubated in 100% humidified air containing 5% CO_2_ at 38.5°C for 42 to 44 h.

### Parthenogenetic activation and *in vitro* embryo culture

COCs were separated from oocytes by treatment with 0.1% hyaluronidase, and the oocytes were activated by stimulation with electrical pulses (120 V for 60 μs) 44 h after *in vitro* culture. Incubation in cytochalasin B for 3 h was performed to prevent discharge of the second polar body. Next, the activated embryos were cultured (approximately 50 activated embryos were transferred to 500 μL of porcine *in vitro* culture medium) in PZM-5 medium supplemented with 0 (control), 1, 5, or 10 μM OA at 38.5°C in 100% humidified air containing 5% CO_2_ in a constant temperature incubator. The 48-h cleavage rate and the blastocyst formation rate were measured on day 2 and day 6, respectively.

### Blastocyst diameter and total blastocyst cell number assays

Images of the blastocysts were taken on day 6 with a microscope, and the diameters of the blastocysts were subsequently analyzed using ImageJ software. To determine the total number of blastocysts present, the blastocysts were collected, transferred to 4% paraformaldehyde and fixed at room temperature for 30 min. Then, the cells were washed with phosphate-buffered saline (PBS) containing 0.1% polyvinylalcohol (PVA) three times for 5 min each. Blastocysts were treated with PBS containing 0.3% Triton X-100 for 30 min, and the nuclei were stained with 10 μg/mL Hoechst 33342 for 10 min. The blastocysts were then fixed to a slide and photographed under a microscope. Finally, the total number of cells in each blastocyst was analyzed using ImageJ software.

### 5-Ethynyl-2′-deoxyuridine analysis

Blastocyst proliferation was determined with a BeyoClickTM EdU-555 cell proliferation detection kit (C0075S; Beyotime, Shanghai, China). 5-Ethynyl-2′-deoxyuridine (EdU) was diluted with cell culture media 1:500 to obtain the EdU working solution. The blastocysts were then incubated with the EdU working solution in an incubator for 2 h. After incubation, the dye was removed by washing the blastocysts with PBS-PVA solution, and the blastocysts were fixed in 4% paraformaldehyde at room temperature for 15 min. The blastocysts were then treated with PBS containing 0.3% Triton X-100 for 15 min and incubated with click reaction solution for 30 min. Finally, the blastocyst nuclei were stained with 10 μg/mL Hoechst 33342 for 10 min. The blastocysts were then fixed on a slide and imaged under a microscope (EdU has a maximum excitation wavelength of 555 nm and a maximum emission wavelength of 565 nm, while Hoechst 33342 has a maximum excitation of 346 nm and a maximum emission of 460 nm). Finally, ImageJ software was used to analyze blastocyst proliferation in each group.

### Apoptosis assay

Apoptosis was determined using the Melun One Step TUNEL Apoptosis Assay Kit (MA0223; MeilunBio, Dalian, China). The collected blastocysts were fixed in 4% paraformaldehyde for 30 min at room temperature. Afterward, the blastocysts were incubated with PBS containing 0.3% Triton X-100 for 15 min at room temperature for permeabilization. Next, the blastocysts were incubated with 50 μL of TdT-mediated dUTP nick end labeling (TUNEL) assay solution at 37°C for 60 min. Subsequently, the nuclei were stained with 10 μg/mL Hoechst 33342 for 10 min. Finally, the blastocysts were fixed on microscope slides and imaged under a microscope (with TUNEL excitation wavelengths ranging from 450 to 500 nm and emission wavelengths ranging from 515 to 565 nm; Hoechst 33342 has a maximum excitation of 346 nm and a maximum emission of 460 nm). The percentage of TUNEL-positive cells was analyzed with ImageJ software.

### EU labeling and staining

A Cell-Light EU Apollo 488 RNA imaging kit (RiboBio, Guangzhou, China) was used to detect the newly synthesized RNA. The 4-cell-stage embryos were incubated with 500 μM EU medium for 5 h and then fixed in 4% paraformaldehyde at room temperature for 30 min. To neutralize the excess paraformaldehyde, 2 mg/mL glycine was added for 5 min of coincubation. Then, the embryos were pretreated with PBS containing 0.5% Triton X-100 for 10 min and incubated with 1×Apolloro staining solution at room temperature for 45 min in the dark. Finally, the cells were stained with Hoechst 33342 for 15 min, mounted, and observed by fluorescence microscopy (EU has an excitation wavelength of 490 nm and an emission wavelength of 520 nm, while Hoechst 33342 has a maximum excitation of 346 nm and a maximum emission of 460 nm) for analysis by ImageJ software.

### Analysis of intracellular reactive oxygen species and glutathione levels

To measure the ROS levels in embryos, the probe DCFH-DA (S0033S; Beyotime, China) was diluted 1:1,000 with PBS-PVA to a final concentration of 10 μM. Then, 4-cell-stage embryos were incubated with the diluted DCFH-DA solution at 37°C for 20 min. To measure the GSH levels in embryos, the dye 4-chloromethyl-6,8-difluoro-7-hydroxycoumarin (C12881, diluted 1:1,000 in PBS-PVA; Thermo Fisher, Waltham, MA, USA) was added to 4-cell-stage embryos for 30 min of incubation. After incubation, the free probe and dye were removed by washing with PBS-PVA, and the fluorescence intensity was determined in real time under a microscope with the same exposure parameters (ROS was measured at an excitation wavelength of 488 nm and an emission wavelength of 525 nm, while GSH has a maximum excitation wavelength of 371 nm and a maximum emission wavelength of 464 nm). The fluorescence intensity of each group of 4-cell-stage embryos was analyzed using ImageJ software.

### Determination of the intracellular total antioxidant capacity

The total antioxidant capacity (T-AOC) of 4-cell-stage embryos was determined using a T-AOC assay kit (BC1315; Solarbio, Beijing, China). Ten microliters of extraction solution was added to forty 4-cell-stage embryos from each group, and the embryos were centrifuged and placed on ice until analysis; after storage, the mixture was preheated at 37°C for 10 min according to the instructions. The assay tubes contained 180 μL of the mixture, 6 μL of the sample supernatant, and 18 μL of distilled water, while the blank tubes contained 180 μL of the mixture and 24 μL of distilled water. Each mixture was allowed to react for 10 min at room temperature after thorough mixing. The absorbance at 593 nm was measured for each group in a 96-well plate (200 μL total volume). Finally, a standard curve was constructed according to the method in the user’s guide, and the absorbance was used to obtain the Fe^2+^ concentration (x). The results were obtained according to the formula for T-AOC (μmol/mL) = 34x.

### Determination of intracellular catalase activity

A CAT activity assay kit (BC0205; Solarbio, China) was used to determine the CAT activity in 4-cell-stage embryos. Briefly, 50 4-cell-stage embryos were collected from each group, and the cells were disrupted via ultrasound and then centrifuged. Next, 10 μL of the supernatant was added to 96-well plates, and 190 μL of working solution was added. The initial absorbance at 240 nm (A1) and the absorbance after 1 min (A2) were recorded.

### Determination of intracellular superoxide dismutase activity

Intracellular SOD activity was examined using a total SOD activity assay kit (WST-8 method) (S0101S; Beyotime, China). Sixty embryos cultured *in vitro* to the 4-cell stage were collected from each group, and the cells were lysed with 90 μL of sample preparation solution after being washed three times with PBS-PVA followed by centrifugation at 4°C for 5 min. Next, 160 μL of prepared WST-8/enzyme working solution and 20 μL of the sample supernatant were added to a 96-well plate, followed by 20 μL of reaction initiation working solution, after which the samples were mixed and immediately incubated at 37°C for 30 min. Finally, the cells were analyzed by enzymatic labeling (Multi-Mode Detection Platform, Spectra Max i3x; Molecular Devices, Shanghai, China) at an absorbance wavelength of 450 nm.

### Determination of the mitochondrial membrane potential

A MitoTracker Red CMXRos kit (C1049B, 50 μg; Beyotime, China) was used to measure the mitochondrial membrane potential. A 200 μM MitoTracker Red CMXRos stock solution was prepared before staining, and the stock solution was subsequently diluted with IVC at a ratio of 1:1,000. The diluted dye solution was made into 10 μL droplets, which were preincubated in an incubator at 37°C. Embryos at the 4-cell stage were incubated with the staining solution at 37°C for 30 min and then washed three times with PBS. MitoTracker Red CMXRos has a maximum excitation wavelength of 579 nm and a maximum emission wavelength of 599 nm. Finally, ImageJ software was used to analyze the fluorescence intensity of each group of embryos.

### Detection of intracellular adenosine 5′-triphosphate levels

Intracellular adenosine 5’-triphosphate (ATP) levels were analyzed using an ATP test kit (S0027; Beyotime, China). Forty 4-cell-stage embryos were taken from each group, washed three times with PBS-PVA, lysed with 80 μL of lysis solution, and then centrifuged at 4°C for 5 min. The ATP test solution was added to the 96-well plate, after which the supernatant was added to the test well. The sample RLU values were then determined using a microplate reader (Multi-Mode Detection Platform, Spectra Max i3x; Molecular Devices, China).

### Western blotting assay

Fifty blastocysts per group were lysed with lysis solution (40% ddH_2_O, 0.5 mM Tris-HCl, 10% sodium dodecyl sulfate [SDS], 50% glycerol, β-mercaptoethanol, and bromophenol blue) at 95°C for 10 min. The protein was separated from each lysed sample via SDS-polyacrylamide gel electrophoresis (PAGE) and transferred to a polyvinylidene fluoride (PVDF) membrane by the wet transfer method. Subsequently, the membrane was blocked with a protein-free rapid blocking solution (PS108P; Epizyme, Shanghai, China) for 15 min and then incubated with primary antibodies against BCL2 associated X protein (Bax) (50599-2-Ig, 1:2,000; Proteintech, Wuhan, China), B-cell CLL/lymphoma 2 (Bcl-2) (12789-1-AP, 1:2,000; Proteintech, China), and β-actin (4970S, 1:1,000; CST, Boston, MA, USA) at 4°C overnight. The membrane was washed three times with tris buffered saline with Tween 20 (TBST) for 10 min each time and then incubated with goat anti-rabbit immunoglobulin G (IgG) (7074S, 1:4,000; CST, USA) at room temperature for 1 h. Finally, the PVDF membrane was washed three times with TBST solution for 10 min each time, and the results were visualized with an enhanced chemiluminescence highsensitivity luminescence detection kit (SQ201; Epizyme, China). Grayscale analysis was performed using ImageJ software.

### RNA extraction and real-time quantitative polymerase chain reaction

Eighty blastocysts were collected from each group and placed in 1.5 mL centrifuge tubes to which 1 mL of TRIzol reagent (Takara, Shiga, Japan) was added to extract RNA. The extracted RNA was then reverse transcribed using MonScript RTIII All-in-One Mix with dsDNase (MR05101M; Monad, Suzhou, China). Real-time quantitative polymerase chain reaction (RT-qPCR) was performed in PCRmax (Eco, Staffordshire, UK) using SYBR Green PCR master mixes (Roche, Basel, Switzerland). RT-qPCR was performed for 40 cycles with the following conditions: 95°C for 10 min, 95°C for 10 s, 60°C for 20 s, and 72°C for 30 s. The data were analyzed by the 2^−ΔΔct^ method using *18S rRNA* as an internal reference gene at the end of the instrument run. The sequences of the primers used in this study are given in Table 1. *SOX2* and *OCT4* ware associated with embryonic pluripotency. *DPPA2* and *ZSCAN4* ware involved in regulating zygotic genome activation.

### Statistical analysis

**Figure 1:**
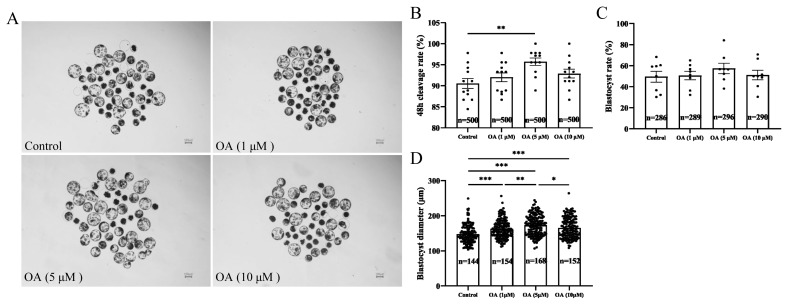
Effects of different concentrations of OA on parthenogenetic embryo development. (A) Representative images of day-6 blastocysts treated with 0 (control), 1, 5, or 10 μM OA. Scale bar, 100 μm. (B) The 48 h cleavage rate of porcine parthenogenetic embryos; “n” represents the number of early embryos counted in each group, and “data points” represents the number of experimental replicates. (C) The blastocyst formation rate of porcine parthenogenetic embryos on day 6; “n” represents the number of early embryos counted in each group, and “data points” represents the number of experimental replicates. (D) The blastocyst diameter of porcine parthenogenetic embryos on day 6; both “n” and “data points” represent the number of early embryos counted in each group. OA, oleanolic acid. Significant differences are indicated by * p<0.05, ** p<0.01, and *** p<0.001.

The data were analyzed by using GraphPad 8.0.2 and R software. The Shapiro-Wilk test was used to test the normality of the data. Student’s t test was used to compare two groups when the data followed a normal distribution, and one-way analysis of variance and Tukey’s multiple comparison test were used to compare multiple groups. The Wilcoxon rank-sum test was used to compare two groups (Figure 4B), and the Kruskal-Wallis rank-sum test and pairwise Wilcoxon signed-rank test were used to compare multiple groups (Figure 1D) when the data did not follow a normal distribution. The results are expressed as the mean±standard error of the mean (SEM), and p<0.05 was considered to indicate a statistically significant difference.

## RESULTS

### Effects of different concentrations of oleanolic acid on early embryonic development in pigs

**Figure 2:**
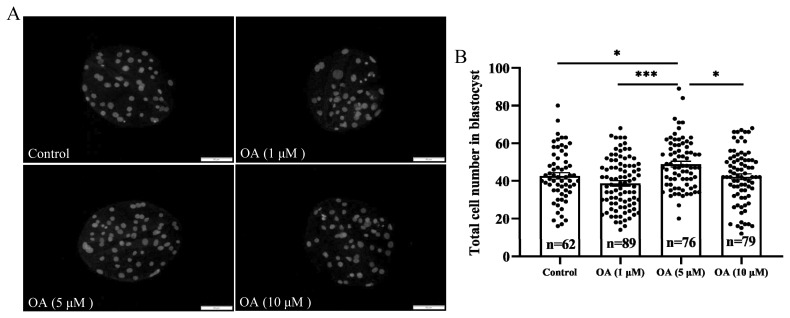
Effect of OA on the total cell count in parthenogenetic embryo blastulae. (A) Representative images of blastocysts stained with Hoechst 33342 on day 6. Scale bar, 50 μm. (B) Total blastocyst cell number on day 6; both “n” and “data points” represent the number of early embryos counted in each group. OA, oleanolic acid. Significant differences are indicated by * p<0.05 and *** p<0.001.

The *in vitro* development of early embryos was observed in each group after treatment with 0 (control), 1, 5, and 10 μM OA. Supplementation with 5 μM OA significantly increased the 48 h cleavage rate of early embryos compared to that in the control group (Figure 1B; p<0.01). The blastocyst formation rates on day 6 after treatment were 49.58%±5.17% (control), 50.64%±4.02% (1 μM OA), 57.39%±4.84% (5 μM OA), and 51.11%±4.57% (10 μM OA) (Figure 1A, 1C). Further analysis revealed that supplementation with 5 μM OA significantly increased the blastocyst diameter (Figure 1D; p<0.001) and the total blastocyst cell number (Figures 2A, 2B; p<0.05). The above results indicated that 5 μM OA treatment had the greatest effect, so 5 μM OA was selected as the optimal supplementation concentration for subsequent experiments.

### Oleanolic acid improves the proliferation of porcine early embryonic cells cultured *in vitro*

**Figure 3:**
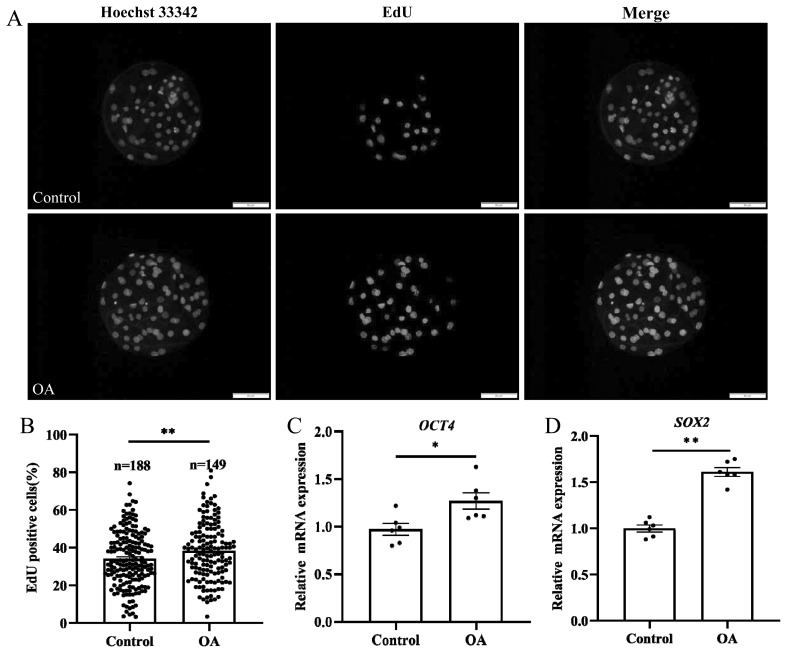
Effects of OA on the proliferation of early porcine embryos. (A) Representative EdU-stained images of blastocysts. Scale bar, 50 μm. (B) Percentages of EdU-positive blastocysts; both “n” and “data points” represent the number of early embryos counted in each group. (C) The relative expression level of *OCT4* mRNA in blastocysts; “data points” represents the number of experimental replicates. (D) The relative expression level of *SOX2* mRNA in blastocysts; “data points” represents the number of experimental replicates. OA, oleanolic acid; EdU, 5-ethynyl-2’-deoxyuridine. Significant differences are indicated by * p<0.05 and ** p<0.01.

To investigate why the total blastocyst cell number increased after OA treatment, cell proliferation in day 6 blastocysts was examined within 2 h. EdU fluorescence staining revealed that the proportion of EdU-positive cells in the control group was significantly lower than that in the OA treatment group (Figures 3A, 3B; control group, 34.10%±1.06% vs OA group, 38.35%±1.26%; p<0.01). RT-qPCR revealed that the mRNA levels of the pluripotency-related genes *OCT4* (Figure 3C; p<0.05) and *SOX2* (Figure 3D; p<0.01) were significantly greater in the OA-treated group than in the control group. The above results indicated that OA could significantly promote blastocyst proliferation and improve blastocyst quality.

### Oleanolic acid inhibits apoptosis in porcine early embryonic cells cultured *in vitro*

**Figure 4:**
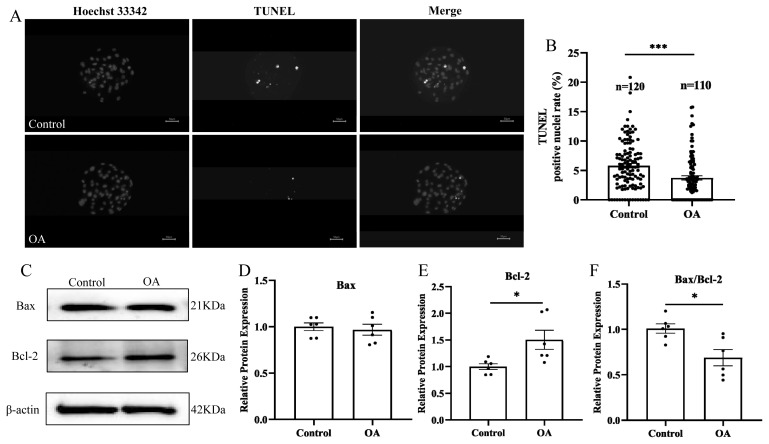
Effects of OA on apoptosis in early porcine embryos. (A) Representative TUNEL-stained images of blastocysts. Scale bar, 50 μm. (B) Rate of TUNEL-positive cells in blastocysts; both “n” and “data points” represent the number of early embryos counted in each group. (C) Western blot analysis of Bax and Bcl-2 expression in porcine parthenogenetic embryos. (D) Relative expression levels of Bax; “data points” represents the number of experimental replicates. (E) Relative expression levels of Bcl-2; “data points” represents the number of experimental replicates. (F) Relative Bax/Bcl-2 ratio; “data points” represents the number of experimental replicates. OA, oleanolic acid; TUNEL, terminal deoxynucleotidyl transferase-mediated dUTP nick end labeling; Bax, BCL2 associated X protein; Bcl-2, B-cell CLL/lymphoma 2. Significant differences are indicated by * p<0.05 and *** p<0.001.

To further investigate why the total blastocyst cell number increased following OA treatment, blastocyst cell apoptosis was examined in early embryos. TUNEL fluorescence staining revealed that the percentage of TUNEL-positive cells in the blastocysts significantly decreased after OA supplementation (Figures 4A, 4B; control group, 5.81%±0.38% vs OA group, 3.73%±0.36%; p<0.001). The Western blot results showed that the Bax protein level was not different (Figure 4D; p>0.05), the Bcl-2 protein level was significantly greater (Figure 4E; p<0.05) and that the Bax/Bcl-2 protein ratio was significantly lower (Figure 4F; p<0.05) in the OA supplementation group than in the control group.

### Oleanolic acid promotes zygotic genome activation in early porcine embryos cultured *in vitro*

**Figure 5:**
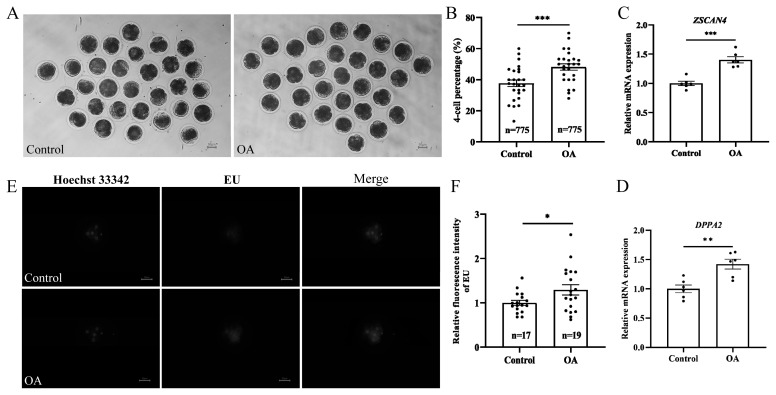
OA affects the process of zygotic genome activation in early porcine embryos. (A) Typical images depicting the effect of OA on the development of porcine embryos 48 h after parthenogenetic activation. Scale bar, 50 μm. (B) Effect of OA on the development rate of porcine 4-cell embryos; “n” represents the number of early embryos counted in each group, and “data points” represent the number of experimental replicates. (C) Relative mRNA expression levels of *ZSCAN4*; “data points” represents the number of experimental replicates. (D) Relative mRNA expression levels of *DPPA2*; “data points” represent the number of experimental replicates. (E) Representative images of EU staining of 4-cell embryos in the control group and OA supplementation group. Scale bar, 50 μm. (F) The relative fluorescence intensity of EU staining of 4-cell embryos; both “n” and “data points” represent the number of early embryos counted in each group. OA, oleanolic acid. Significant differences are indicated by * p<0.05, ** p<0.01, and *** p<0.001.

The 4-cell stage is a critical period for porcine zygotic genome activation. After *in vitro* culture for 48 h, the proportion of embryos that developed into 4-cell embryos was significantly higher in the OA-treated group than in the control group (Figures 5A, 5B; control group, 37.67%±2.17% vs OA group, 48.19%±2.04%; p<0.001). The RT-qPCR results showed that the mRNA levels of genes related to zygotic genome activation, namely, *ZSCAN4* (Figure 5C; p<0.001) and *DPPA2* (Figure 5D; p<0.01), were significantly greater than in the OA-treated group than in the control groups. Furthermore, the EU staining results showed that the fluorescence intensity was significantly lower in the control group than in the OA group (Figures 5E, 5F; p<0.05), indicating a significant increase in the amount of de novo RNA in the OA group.

### Oleanolic acid alleviates oxidative stress in early porcine embryos cultured *in vitro*

**Figure 6:**
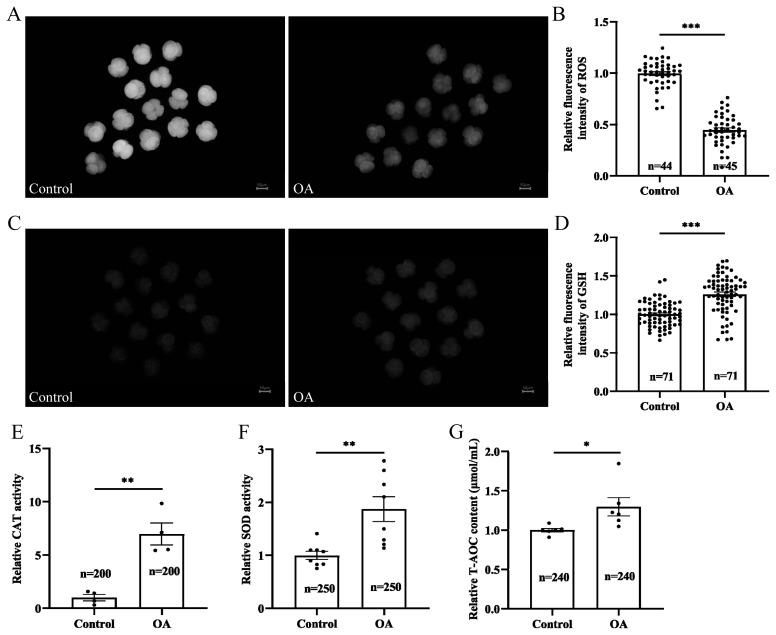
Effects of OA on the antioxidant capacity of early porcine embryos. (A) Representative fluorescence images of ROS staining in 4-cell-stage embryos. Scale bar, 50 μm. (B) The relative fluorescence intensity of ROS in 4-cell embryos; both “n” and “data points” represents the number of early embryos counted in each group. (C) Representative fluorescence images of 4-cell-stage embryos stained for GSH. Scale bar, 50 μm. (D) The relative fluorescence intensity of GSH after staining in 4-cell embryos; both “n” and “data points” represents the number of early embryos counted in each group. (E) The relative CAT activities in 4-cell embryos in the control group and OA supplementation group; “n” represents the number of early embryos counted in each group, and “data points” represents the number of experimental replicates. (F) The relative SOD activities in 4-cell embryos from the control group and OA supplementation group; “n” represents the number of early embryos counted in each group, and “data points” represents the number of experimental replicates. (G) The relative T-AOC of 4-cell-stage embryos in the control group and OA supplementation group; “n” represents the number of early embryos counted in each group, and “data points” represents the number of experimental replicates. OA, oleanolic acid; ROS, reactive oxygen species; GSH, glutathione; CAT, catalase; T-AOC, total antioxidant capacity. Significant differences are indicated by * p<0.05, ** p<0.01, and *** p<0.001.

The above results indicated that OA improved the developmental capacity of early porcine embryos, which we speculated might be due to the improved antioxidant capacity of early embryos induced by OA; therefore, we examined the levels of ROS and GSH in early embryos at the 4-cell stage. The results showed that the intracellular level of ROS in embryos supplemented with OA decreased to 0.45±0.02 relative to that in the control group (Figures 6A, 6B; p<0.001). Moreover, the intracellular fluorescence intensity of GSH in the embryos in the OA-treated group was 1.26±0.03 times greater than that in the control group (Figures 6C, 6D; p<0.001). Further analysis revealed that the CAT and SOD enzyme activities were significantly greater in the OA group than in the control group (Figures 6E, 6F; p<0.01). Moreover, the T-AOC in the OA group was also significantly greater than that in the control group (Figure 6G; p<0.05). These results indicate that OA can indeed effectively enhance the antioxidant capacity of early embryos.

### Oleanolic acid improves mitochondrial function in early porcine embryos cultured *in vitro*

**Figure 7:**
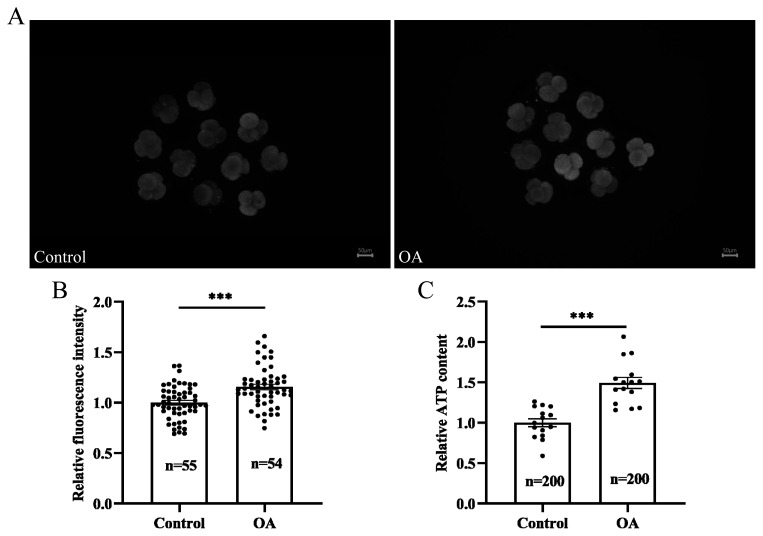
Effects of OA on mitochondrial function in early porcine embryos. (A) Representative fluorescence images of mitochondrial membrane potential staining in embryos at the 4-cell stage in the control group and OA supplementation group. Scale bar, 50 μm. (B) Relative fluorescence intensities indicating the mitochondrial membrane potential in 4-cell embryos; both “n” and “data points” represent the number of early embryos counted in each group. (C) The relative levels of ATP in 4-cell embryos; “n” represents the number of early embryos counted in each group, and “data points” represents the number of experimental replicates. OA, oleanolic acid; ATP, adenosine 5’-triphosphate. Significant differences are indicated by *** p<0.001.

The ROS, when present in excess, cause oxidative stress, which results in mitochondrial dysfunction. As shown in Figure 6, OA supplementation alleviated oxidative stress in early-stage porcine embryos cultured *in vitro*; therefore, we further investigated mitochondrial function. Fluorescence staining revealed that the mitochondrial membrane potential was significantly greater in the OA-treated group than in the control group (Figures 7A, 7B; p<0.001). In addition, the level of intracellular ATP significantly increased after OA supplementation (Figure 7C; p<0.001). The above results indicated that OA improved mitochondrial function in early porcine embryos.

## DISCUSSION

Oxidative stress is a key factor affecting the quality of embryos cultured *in vitro* [20]. This may be due to the accumulation of metabolic waste and the relative lack of free radical scavengers in *in vitro* cultured embryos. OA is a natural antioxidant with potent free radical scavenging activity [21]. In this study, we found that 5 μM OA effectively promoted early embryonic development competence and improved embryo quality *in vitro*.

Cell proliferation is closely related to embryo quality. Therefore, the proliferation of blastocyst cells was evaluated in this study. The results showed that the proportion of EdU-positive cells was significantly greater in the OA-treated group than in the control group. Thus, OA could effectively promote the proliferation of blastocyst cells. OCT4 is a marker for totipotent cells and plays an important role in inner cell mass formation and cell proliferation in pigs [22]. Another study showed that knockdown of *SOX2* affects the formation of the inner cell mass [23]. In this study, the mRNA levels of both *OCT4* and *SOX2* were significantly higher in the OA treatment group, suggesting that OA improves early embryonic development in pigs.

Apoptosis is a fundamental biological phenomenon of cells. However, unprogrammed cell death can occur during the *in vitro* culture of embryos due to environmental stress and other reasons, which ultimately affects the developmental quality of early embryos [24]. The study have shown that OA can reduce oxidized low-density lipoprotein-induced apoptosis in human umbilical vein endothelial cells through inhibition of the LOX-1/ROS/HIF-1α pathway [25]. The results of TUNEL fluorescence staining showed that the percentage of apoptotic blastocysts in the OA group was significantly lower than that in the control group. The Bcl-2 family of proteins are key regulators of apoptosis, with both antiapoptotic (Bcl-2, Bcl-XL, and others) and proapoptotic (Bax, Bak, and Bok, and others) members [26]. Bcl-2 is expressed at high levels in high-quality oocytes and embryos, whereas Bax is expressed at high levels in naked oocytes or poor quality embryos [27]. In the present study, the Bax/Bcl-2 ratio was lower in the OA group than in the control group. This suggests that OA may improve the quality of early porcine embryos by promoting the proliferation of blastocyst cells and reducing their apoptosis.

The 4-cell stage is the main period of zygotic genome activation in pigs, as before this point, embryonic development is mainly under the control of maternal mRNA [28]. Zygotic genome activation is a gradual transcriptional activation process that may fail if ROS attack occurs in the external environment during this period [29]. *ZSCAN4* is considered a marker gene for zygotic genome activation [30]. A decrease in the expression of *ZSCAN4* affects the development of bovine embryos from the 16-cell stage to the blastocyst stage [31]. *DPPA2* directly regulates the transcription factor *Dux* and thereby promotes the activation of the zygotic genome [32]. The results here showed that the rate of developmental arrest in 4-cell embryos was significantly reduced in the OA-treated group, and the levels of *ZSCAN4* and *DPPA2* mRNA were significantly increased. Furthermore, nascent mRNA expression was also significantly increased in the OA group, as shown by the EU staining results. The above results indicated that OA promoted zygotic genome activation during early porcine embryonic development and reduced blockade of 4-cell embryo development.

Many studies have shown that OA can alleviate oxidative stress by scavenging the excess ROS produced [33–35]. In the present study, we found that the fluorescence intensity of ROS significantly decreased in the OA group while the fluorescence intensity of GSH significantly increased, which was consistent with previous findings [36]. SOD and CAT are important cellular antioxidants [37], as SOD reacts with the superoxide anion (O_2_·^−^) to produce hydrogen peroxide (H_2_O_2_) and oxygen (O_2_) [38], and CAT reduces H_2_O_2_ [39]. Our results showed that the SOD and CAT enzyme activities were significantly increased after OA supplementation. These results suggest that OA can improve early porcine embryo development *in vitro* by increasing the embryonic antioxidant capacity and alleviating oxidative stress.

Mitochondria are important organelles involved in a variety of cellular activities in early mammalian embryos, primarily regulating energy and nutrient metabolism. Increasing evidence indicates a close link between mitochondria and oxidative stress [40]. Mitochondrial dysfunction can cause an excess of ROS to be produced, resulting in cellular damage that affects embryonic development [41]. When intracellular ROS levels are high, the mitochondrial membrane potential is reduced [42]. Previous studies have shown that OA scavenges the excess ROS in early embryonic cells, which may further improve mitochondrial function. As we expected, in the present study, the mitochondrial membrane potential and ATP levels were significantly greater in the OA group than in the control group. These results indicate that OA may improve mitochondrial function. However, the possible mechanism by which oxidative stress affects mitochondrial function still needs further investigation.

This study exclusively investigated the impact of OA on the development of parthenogenetically activated embryos and did not establish that OA can enhance the development of IVF embryos. However, other studies have indicated that asiatic acid, a pentacyclic triterpenoid compound similar to OA, not only enhances the development of parthenogenetically activated embryos but also increases the blastocyst formation rate of both IVF and SCNT embryos [11]. Other antioxidants, such as resveratrol [43] and maslinic acid [44], have also been shown to increase the developmental potential of both parthenogenetically activated and IVF embryos. Therefore, it is reasonable to hypothesize that OA, as an antioxidant, promotes IVF embryo development.

## CONCLUSION

In conclusion, the present experimental study demonstrated that supplementing parthenogenetically activated porcine embryos with an appropriate concentration (5 μM) of OA during *in vitro* culture could effectively enhance their antioxidant capacity, inhibit the occurrence of apoptosis, and improve mitochondrial function, which could contribute to enhancing the developmental potential of early embryos *in vitro*.

## Figures and Tables

**Table 1:** Sequences used for real-time quantitative polymerase chain reaction

Genes	Primer sequence (5′-3′)	Accession
*18S rRNA*	F-GCCCGAAGCGTTTACTTTGA	NR_046261.1
	R-CCGCGGTCCTATTCCATTATT	
*OCT4*	F-GTGAGAGGCAACCTGGAGAG	NM_001113060.1
	R-TCGTTGCGAATAGTCACTGC	
*SOX2*	F-AAGAGAACCCCAAGATGCACAACT	NM_001123197.1
	R-GCTTGGCCTCGTCGATGAAC	
*DPPA2*	F-CCGTTCCTGCTTCTGTTGAGACC	XM_003358822.4
	R-GGCGAACCCAACCTTCTGTATCTG	
*ZSCAN4*	F-GCCCAGAAAGTCTTCCCATGTGAG	XM_021097584.1
	R-GCCTCTCATCATTGTGTCTCCTCTG	
